# Stimulated enrichment of *Clostridium difficile* specific IgA in mature cow's milk

**DOI:** 10.1371/journal.pone.0195275

**Published:** 2018-04-25

**Authors:** Christiane Schmautz, Maria Hillreiner, Ines Ballweg, Michael W. Pfaffl, Heike Kliem

**Affiliations:** Chair for Animal Physiology and Immunology, Technical University of Munich (TUM), Freising, Germany; University of Illinois, UNITED STATES

## Abstract

Cow milk products enriched with *Clostridium difficile* (*C*. *diff*.) specific IgA are possible alternative therapeutics against *C*. *diff*. associated diarrhea. A persistently high level of *C*. *diff*. specific IgA in mature milk triggered by continuous immunizations of dairy cows against *C*. *diff*. was hypothesized. Nine *Brown Swiss* cows were repeatedly vaccinated against *C*. *diff*. and divided into low responder (LR) and high responder (HR) cows, as measured by their production of anti-*C*. *diff*. specific IgA in milk (threshold: 8.0 μg anti-*C*. *diff*. specific IgA/mL on average). Total and *C*. *diff*. specific IgA were quantified in bovine milk and blood using a sandwich ELISA. Important milk production factors were analyzed per lactation stage. Milk yield, milk fats and proteins were significantly different (P < 0.05) in the early lactation stage when the treated with the untreated cows (n = 30) were compared. In contrast to the "before treatment control" values, the HR's milk anti-*C*. *diff*. IgA was approximately 80% higher at any lactation stage, and the HR's total milk IgA increased up to 72% in the late lactation stage. The LR's total milk IgA differed from the baseline by roughly 47% only in the late lactation stage. The total and specific IgA contents in milk were more influenced by the anti-*C*. *diff*. immunizations than in blood. The correlations between anti-*C*. *diff*. specific IgA, total IgA and the main production factors in milk were classified as weak (*I r I < 0*.*5*), except for the close relation of anti-*C*. *diff*. specific IgA and total IgA (*r = 0*.*69*). To conclude, a sustainable *C*. *diff*. specific IgA enrichment in milk can be achieved by continuous immunization of dairy cows, provided a potent and well-formulated anti-*C*. *diff*. vaccine is given to dairy cows preselected due to their proven anti-*C*. *diff*. receptivity.

## Introduction

Milk is a foodstuff of high biological value in mammalian nutrition. In addition to the species-adapted proportions of the macronutrients, lactose, milk fat and protein, milk delivers various health-promoting constituents. The main immune-relevant components in milk, and especially in colostrum, are immunoglobulins (Igs). Depending on species, breed, age, stage of lactation and health status, varying proportions of the different immunoglobulin (Ig) classes are inherent in mammary secretions [1]. In humans, the predominant Ig class is IgA, whereas IgG has the largest share of colostral and milk antibodies in cattle [2]. Concentrations of 20–200 mg IgG/mL milk, being dominated by the principal subclass IgG1, are found in bovine colostrum. To ensure the supply of calves with essential antibodies, colostrum of the dam contains remarkably higher amounts of IgG, which comprise 70–80% of total protein in contrast to its lower remaining level of 1–2% in mature milk [3].

In line with passive immunization (IM) of bovine neonates, the concept of immune milk for human health was developed as early as in the mid-20^th^ century. Petersen & Campbell (1955) pointed out "*the use of protective principles in milk and colostrum in prevention of disease in man and animals"* [4]. They had laid the cornerstone for the utilization of minor milk components in the health care and dairy industries, first commercialized by Ralph Stolle (http://www.smbimilk.com/) [5]. IM procedures with polyvalent vaccines showed that the specificity of lacteal antibodies can be influenced [6]. The volume of antigen-specific lacteal antibodies would grow if pregnant cows were vaccinated with certain antigens at the end of their gestation so they could benefit from the natural accumulation of Igs in bovine colostrum. A hyperimmune bovine colostrum (HBC) was generated in this way, in retrospect mainly for its application in the prevention or treatment of animal and/or human enteric diseases [7, 8]. For instance, cryptosporidiosis, shigellosis, infantile rotavirus gastroenteritis, enterotoxigenic *E*. *coli* caused diarrhea and *Clostridium difficile* (*C*. *diff*.) associated diarrhea (CDAD) were successfully treated with nutraceuticals like HBC or purified derivate products [9]. The therapeutic activity of such an orally administered bovine Ig concentrate depends directly on the survival of the included antigen-specific IgG during its passage through the recipient's intestinal tract. In this context, Kelly et al. (1997) found that the consumed bovine colostral IgG undergoes partial degradation while retaining antigen-specific neutralization capacity [10]. More than 10% of IgG was recovered in stools from infants treated with an Ig concentrate [11]. Resistance to proteolytic digestion depends on the isotype of antibody. The secretory IgA (sIgA), naturally occurring in mucosal sites, resists proteolytic digestion better than IgG due to the protection of sIgA by the linked secretory component [12]. Bovine colostrum and milk comprise sIgA in concentrations of 1–6 mg/mL and 0.05–0.1 mg/mL, respectively [3]. However, the average 18% sIgA of total milk Igs provide a powerful antigen-specific neutralization capacity [1]. Exemplarily, polymeric IgA was revealed as superior to monomeric IgA and IgG in preventing the damaging effects of *C*. *diff*. toxin A *in vitro* [13]. Furthermore, sIgA does not encourage inflammatory processes as it is neither able to opsonize nor to activate complement, unlike IgG [14]. Overall, the features of sIgA described above are advantageous for the complementary immunotherapy of CDAD, as demonstrated by using a particular sIgA-enriched bovine whey protein concentrate in the hamster model and in a clinical trial performed with human beings [15, 16].

*C*. *diff*. is a gram-positive, anaerobic, spore-forming and rod-shaped bacterium ubiquitously present in the environment. It is present as a normal, but minor inhabitant of the intestinal microflora of healthy human adults and animals [17]. Toxigenic strains of *C*. *diff*. produce two exotoxins, named toxin A (TcdA) and B (TcdB), which affect the intestinal integrity by destroying the cytoskeleton of the enterocytes. Essential prerequisites for *C*. *diff*. infections (CDI) in humans are a weakened immune system (IS) due to advanced age, co-morbidity, antibiotic therapy and residence in nursing homes and hospitals with increased *C*. *diff*. spore load. Antibiosis disturbs the indigenous flora of the gastrointestinal tract and offers *C*. *diff*. a niche for adherence and vegetation. The clinical picture of CDI varies from asymptomatic carriage to life-threatening pseudomembranous colitis or toxic megacolon [18, 19]. 20–35% of patients experience a relapse after standard antibiotic treatment with the commonly used antibiotics vancomycin or metronidazole [20]. Furthermore, hypervirulent *C*. *diff*. strains like BI/NAP1/027, which produce a third, binary toxin, have become more prevalent worldwide since the CDI epidemics in North America and Europe (2003 & 2004) [21]. The increased incidence and severity of this disease invite alternative treatment approaches. Oral immunotherapy with *C*. *diff*. specific sIgA from bovine immune milk would not eradicate *C*. *diff*. right now, but could serve as a possible supplementary approach to antibiotic therapy to prevent CDAD.

As the history of immune milk production has shown, repeated exposure of dairy cows to the selected pathogen would be necessary to produce of cow's milk enriched with *C*. *diff*. specific antibodies. The present study builds on the promising approach regarding the IM procedure utilized by van Dissel et al. in 2005 [15]. In contrast to the previously tested efficacy of the immune whey protein concentrate on CDAD prevention [15], the aim of this study was to focus on the quantities of anti-*C*. *diff*. sIgA that can be steadily produced in milk. It was hypothesized that a persistently high level of *C*. *diff*. specific IgA in mature milk would be ensured by continuous triggering of the dairy cows with the virulent factors of *C*. *diff*. until completion of lactation. Therefore, specific IM procedures were carried out and the immune response of the vaccinated cows was closely supervised.

## Materials and methods

### Animals

The government of Upper Bavaria gave its approval to conduct the animal trial (AZ 55.2-1-54-2532.6-17-2012). Nine healthy and primiparous *Brown Swiss* cows were used for the IM study. They were allocated to a separate byre with tethering for easier operation. The early lactating cows with 28 ± 4 milking days (DiL) on average produced 21.3 ± 1.5 liters milk per day. After the lactation time ended, they were slaughtered and their carcasses fully disposed of without touching the food chain. The reference dairy cattle group was provided by the Veitshof research station of the Technical University of Munich at Freising, Germany, where they were housed in a freestall barn. Thirty *Brown Swiss* cows in the first or second lactation were selected and assigned to an early, mid- or late lactation group depending on their lactation state. Throughout the entire study period, all cows had water access *ad libitum* and were fed a daily basic ration that consisted of 22 kg of corn silage (33% dry matter), 10 kg of grass silage (40% dry matter) and 2 kg of hay. For energy balance, they received 2 kg of high-protein crushing rape and soy (deuka Kompopur 404; Deutsche Tiernahrung Cremer, Düsseldorf, Germany) in their diet. Their mineral balance was achieved by supplementation of 125 g of mineral mix (Josera, Kleinheubach, Germany). Dairy cow performance requirements were met by giving them 0.5 kg of concentrate (deuka MK 194-UDP; Deutsche Tiernahrung Cremer) per liter of milk delivered.

### Vaccination plan

Before launching the experiment, the animals assigned for treatment were allowed a five-day acclimatization period in the cowshed environment. After a health check by a veterinarian, only those cows showing no signs of illness were vaccinated. The same procedure was carried out prior to every further vaccination. Additionally, the health status of the animals was always audited on the first day after the vaccination. Samples associated with sick animals were excluded from the analysis. The *vaccine* consisted of whole *C*. *diff*. cells (strain VPI 10463) formaldehyde-inactivated, and the partly disabled TcdA and TcdB prepared from the *C*. *diff*. culture filtrate. Two *MucoCD-I* vaccine batches (A and B) for injection, including different toxoid A and B proportions in relation to the respective toxins, and the vaccine *MucoCD-N* for nasal (N) application were provided by IDT Biologika (Dessau-Roßlau, Germany). The *vaccination schedule* implemented is summarized in Table 1. The cows were immunized on the N route biweekly. The periodically administered N vaccinations were complemented with *MucoCD-I* given percutaneously (PC) close to both supramammary lymph nodes (SLN) every four weeks and subcutaneously (SC) or rather intracutaneously (IC) on the lateral torso (by several punctures into a 20 x 15 cm shorn area caudal of the scapula) at different points in time from treatment week (TW) 17. Because of incompatible concentrations of intact toxins TcdA and TcdB existing in vaccine batch A, the immune stimulation was restricted to the application of *MucoCD-N* from TW 3 up to TW 15, the end of the first IM period. The second treatment period started with the application of vaccine batch B. Its administration was modified by replacing the SC injection with the intracutaneous (IC) one due the improved efficacy of this administration route. To stimulate a long-lasting antibody response, incomplete Freund’s adjuvant (iFA) was additionally injected in TW 31. The 31-week vaccination period was accompanied by intensive surveillance of its physiological effects.

**Table 1:** Immunization schedule.

TW		1	3	5	7	9	11	13	15	17	19	21	23	25	27	29	31
*MucoCD-N* vaccine		X	X	X	X	X	X	X	X	X	X	X	X	X	X	X	X
*MucoCD-I* vaccine batch			A							B		B		B	B	B	B
*+* iFA																	X
vaccine application	*PC*		X							X		X		X		X	
	*SC*											X					
	*IC*														X		X

During the 31-week treatment period, anti-*C*. *diff*. vaccinations were conducted only in the indicated TWs. The types of vaccine application were marked by crosses. The N vaccination with 2 mL *MucoCD-N* vaccine to each nostril was regularly given bi-weekly. PC close to the SLN, 2 mL of *MucoCD-I* vaccine (A or B) were injected in TWs 3, 17, 21, 25 and 29. On the SC route, 4 mL of *MucoCD-I* vaccine (B) were administered only once in TW 21 and on the IC route in TW 27. Additionally, 2 mL of iFA combined with 2 mL *MucoCD-I* vaccine (B) were IC applied in TW 31.

### Rationale of vaccination sites

The *N IM* was administered by injecting 2 mL of *MucoCD-N* vaccine in each nostril with a needle-free syringe. The direct contact of antigen with the N mucous membrane should stimulate the NALT (nasal associated tissue), a lymphoid tissue located in the olfactory organ and part of the mucosal IS, being closely connected with other mucosal tissues of the body. The local administration of the anti-*C*. *diff*. vaccine close to both SLN on the *PC route* should lead the antigen to the SLN draining the udder by way of the afferent lymphatics. Ensuring the encounter of antigen with naïve lymphocytes, an augmented egress and settlement of specific antibody producing cells (APCs) in the mammary gland were expected. *SC or IC administrations* of antigen caudal of both scapulae should trigger the systemic humoral immunity against *C*. *diff*. and its toxins by increasing the specific serum antibodies. Naïve lymphocytes of peripheral lymph nodes are steadily on an inter-nodal journey in search for their target antigen, thus increasing the probability of an encounter. After lymphocyte activation by antigen contact, their homing is followed by specific antibody production, and the blood becomes enriched with them [22, 23].

### Milk yield and sampling

Milk yield and its composition were monitored in all cows with at least 300 DiL until lactation ended. The quantities of milk gathered were calculated as energy corrected milk (ECM) using the formula: ECM = milk yield-kg*[(0.38*milk fat %) + (0.21*milk protein %) + 1.05] / 3.28 [24].

#### Treated cows

Milk yield was recorded every two weeks with a TRU-Test milk meter (Lemmer-Fullwood, Lohmar, Germany). Once a week, half a liter of milk per cow was deposited during the morning milking. Of this quantity, a 50 mL aliquot containing a preservative (acidiol) was taken to analyze the major milk components, including somatic cell counts (SCC). This milk testing was carried out by the Milchprüfring Bayern Association (Wolnzach, Germany). The milk fats and proteins were measured based on their infrared absorption behavior with the MilkoScan-FT-6000 device (FOSS, Hamburg, Germany). Afterwards, the data were evaluated after transformation using the Fourier method. SCC in milk were analyzed with an optical fluorescent technique (*Fossomatic-FC* device, FOSS, Hamburg, Germany). Milk for measurement of the Ig concentrations was collected weekly in the first two months of treatment and every two weeks subsequently. Two 10 mL samples of the residual 450 mL milk sample per cow were defatted by centrifugation (4,000 xg, 4°C, 15 min). Skimmed milk was aliquoted in 2-mL tubes and stored at -20°C until further processing. Milk samples of cows suspected of mastitis were bacteriologically examined by the Central Bavarian Animal Health Service Association (TGD Bayern, Grub, Germany).

#### Control cows

Milk yield and proximates were captured during the course of the regular analysis for the Bavarian Dairy Herd Improvement Association (LKV Bayern e. V., Munich, Germany). Milk of the selected control cows was collected only once to analyze the IgA content.

### Blood sampling

Blood of the treated cows to measure IgA concentration was collected simultaneously to the milk samples. After morning milking, blood of the jugular vein was collected with a 9 mL EDTA pre-coated vacuette (Greiner Bio-One GmbH, Frickenhausen, Germany) and subsequently stored in ice. Additional 100 μL of 0.3 M EDTA [33.5 g Titriplex III (Merck KgA, Darmstadt, Germany) dissolved in 300 mL bi-distillated water and supplemented with 1% acetylsalicylic acid (Merck KgA, Darmstadt, Germany)] were added to guarantee the stabilization of the bovine blood. After centrifugation (2,000 xg, 4°C, 15 min), the plasma was divided into 2 mL aliquots and stored at -20°C.

### *C*. *diff*. specific IgA

*C*. *diff*. specific IgA in cow's milk and blood was quantified with a sandwich ELISA. The reagents and buffers used were: Coating buffer (35 mM NaHCO_3_, 15 mM Na_2_CO_3_x10H_2_O, pH 9.6), wash buffer (137 mM NaCl, 8 mM Na_2_HPO_4_x2H_2_O, 1.5 mM KH_2_PO_4_, 2.7 mM g/l KCl, 0.1% Tween 20), PBST (0.2 mM NaH_2_PO_4_xH_2_O, 1.2 mM Na_2_HPO_4_x2H_2_O, 0.05% Tween 20, pH 7.4), blocking buffer (2% gelatin in PBST), dilution buffer (0.2% gelatin in PBST), 3,3’,5,5’-tetramethylbenzidine (TMB) enzyme substrate mix and stop solution (2 M H_2_SO_4_). First of all, 96-well plates (Nunc MaxiSorp™, Sigma-Aldrich Chemie GmbH, Munich, Germany) were pretreated with 100 μL/well of *C*. *diff*. cells included in coating buffer (2.0x10^8^ cells/mL, IDT Biologika, Dessau, Germany). The coating lasted 2 h at 70°C and then overnight at 4°C. The addition of 200 μL of blocking buffer for 1 h at 37°C blocked the overlay. After its decantation, the wells were rinsed four times with wash buffer. *C*. *diff*. specific IgA (1.76 mg/mL, MucoVax b. v., Leiden, Netherlands) used as standard was diluted within the range of 62.5 to 4,000 ng/μL with dilution buffer. The samples for testing, skimmed milk or blood serum were diluted 1:10 so they would fall within the range of the standard. Seven dilution stages of the standard, a blank (pure dilution buffer), the samples and intra-assay controls additionally used were applied to the pre-coated plate (100 μL/well) in duplicates for incubation lasting 1.5 h at 37°C. The incubation was completed by repeated washing and the subsequent addition of 100 μL of secondary antibody (1:70,000 diluted HRP conjugated sheep anti-bovine IgA (Bethyl Laboratories, Inc.; Montgomery, TX 77356, USA) to the processed wells. After 1.5 h of exposure at 37°C and protected from light, the wells were rinsed four times and then filled with 150 μL of the TMB substrate mix. The enzymatic reaction took place on a wave platform shaker and in the dark within 40 min at RT. It was terminated by pouring 50 μL of stop solution per well. During the subsequent 30 min, absorbance was evaluated at 450 nm with the photometric instrument (microplate reader Sunrise™, Tecan Group Ltd., Männedorf, Switzerland). The calculation of *C*. *diff*. specific IgA quantities in unknown samples is based on the standard curve generated by the Magellan™ V6.6 reader software (Tecan Group Ltd., Männedorf, Switzerland).

### Total IgA

The bovine IgA ELISA kit (Cat. No. E10-121) provided by Bethyl Laboratories, Inc. was used for the quantitation of total IgA in cow's milk and blood. Other test components used and separately supplied were 96-well plates and the ensuing buffer preparations: Coating buffer (0.05 M carbonate-bicarbonate, pH 9.6), solution for washing, blocking and as diluent (50 mM Tris, 0.14 M NaCl, 0.05% Tween 20, pH 8.0), enzyme substrate (TMB) and stop solution (0.18 M H_2_SO_4_). The ELISA test was carried out according to the manufacture’s recommendations. Finally, the absorbance of each sample was measured within 30 min at 450 nm using the plate reader (Sunrise™).

### Statistics

The MIXED procedure model in SAS/STAT® 9.22 (2010 SAS Institute Inc., Cary, NC, USA) was used to find treatment effects and differences among groups during the treatment period. The computational procedure could only work when the convergence criteria were met, which were evaluated following the log-likelihood calculation. The results were expressed as estimated least square means (LSM) with associated standard errors (SD). The Pearson product moment correlation coefficients (*r*) were calculated by means of SigmaPlot 11.0 (2008 Systat Software, Inc., San Jose, CA 95131, USA). The significance level was set at P < 0.05. Results with P < 0.01 were defined as highly significant.

## Results

### Note

As a preliminary remark, it has to be pointed out that all the data surveyed for the treated cows were evaluated and stringently divided into ‘low responder’ (LR) and ‘high responder’ (HR) cows. The enrichment of *C*. *diff*. specific IgA in milk was done by repeated IM. In particular, the overall view of this parameter had shown that cows were differently sensitive towards the given antigen. Based on their immunological reactivity, the treated cows were assessed as LR or HR by the fixed threshold of 8.0 μg/mL *C*. *diff*. specific IgA in milk. For this purpose, the total average of *C*. *diff*. specific IgA in milk, relating to all measurement points of the entire treatment period, was calculated per cow.

### Animal health

Table 2 summarizes all diseases of the vaccinated cows occurring at the start and during the treatment period. The TW, in which the different diseases emerged, are quoted therein. No health impairments of the control cows to the different dates of sampling were noticed.

**Table 2:** Diseases of treated cows and the TW per vaccinated cow in which diseases emerged.

Diseases	Treated cows								
	LR-2	LR-3	LR-6	LR-7	HR-1	HR-4	HR-5	HR-8	HR-10
Mastitis	1, 8					0			0, 16, 26
Bursitis				1			1		
Lameness			2, 3		0, 1				
Fever				1, **3**	1	**3**	**3**, **4**	**3**	**3**, 23
Udder swelling on IM side	**3**, **4**	**3**, **4**		**3**, **4**		**3**, **4**	**3**, **4**	**3**, **4**	**3**
Hemolysis		**4**		**3**, **4**			**4**		
Ketosis							**4**		

TWs with side effects associated with the one-time PC administered vaccine batch A are in bold.

LR = low responder cow numbers 2, 3, 6, 7; HR = high responder cow numbers 1, 4, 5, 8, 10.

Bursitis, and sometimes lameness and mastitis, were diagnosed mainly when treatment began. These diseases conceivably arose due to local transition of the animals in question to the new byre environment. Because of its impaired health, cow HR-1 did not receive the first N anti-*C*. *diff*. vaccination in TW 1. Clinical signs like fever, udder swelling on the IM side, hemolysis and ketosis, but also reduced feed intake and rumen turnover were deemed to be the cause of the high dosage of the *C*. *diff*. toxins delivered with *MucoCD-I* batch A in TW 3. Cow LR-6 was not immunized in TW 3 owing to its persisting lameness. Therefore, no treatment-related signs could develop hereinafter.

### Milk yield & proximates

With the onset of lactation, control cows produced 30.6 ± 1.3 kg milk per day on average, at least 5 kg/d more milk than the treated cows (Table 3). In association with the adverse health effects caused by the first PC vaccination, a reduction in emitted milk volume was noted for the treated cows in early lactation. When lactation proceeded, significant differences in dairy production no longer existed among the groups, and all cows had persistent milk production within the range of 27 kg/d to 32 kg/d. The daily averages of the treated cows' milk production increased significantly by 22% in the mid-lactation of the LR group and by 14% in the late lactation of the HR group, both with regard to the initial 100 DiL. The concentrations of milk fats and proteins of the control group were 1.29 ± 0.1 kg/d and 1.00 ± 0.1 kg/d, respectively, in early lactation. In this lactation stage, these milk components were significantly higher than those of the treated groups by at least 26% for milk fats and 22% for milk proteins. These group differences ceased in the following lactation stages due to the increased ECM yields of both treated groups. Moreover, the percentages of the major milk components did not differ among all examined groups at any time. Regarding the SCC in milk, the threshold of 10^5^ SCC/mL was exceeded only by the HR group in the mid-lactation stage (Table 3). Several times during this lactation period, mainly between TW 16 and 20, milk of cow HR-8 contained up to 240,000 SCC/mL, and a peak value of 540,000 SCC/mL was determined in the milk of cow HR-10. In sharp contrast, cows of the LR group had produced milk poor in SCC after the first 100 DiL. Control cows approached the threshold with a mean number of 98,100 SCC/mL in early lactation, because about 40% of the control cows had more than 150,000 SCC/mL during this period.

**Table 3:** Milk yield and proximates related to different groups and lactation stages.

lactation stage	group	ECM yield (kg/d)			milk proteins (kg/d)			milk proteins (%)			milk fats (kg/d)			milk fats (%)		SCC (x1,000/mL)		
		LSM	*SD*		LSM	*SD*		LSM	*SD*		LSM	*SD*		LSM	*SD*	LSM	*SD*	
early	control	30.6	*1*.*3*	^a^	1.00	*0*.*05*	^a^	3.3	*0*.*2*		1.29	*0*.*10*	^a^	4.2	*0*.*3*	98.1	*28*.*9*	
	LR	23.7	*0*.*9*	^b^	0.76	*0*.*03*	^b^	3.2	*0*.*3*		0.96	*0*.*07*	^b^	3.8	*1*.*3*	60.4	*21*.*6*	
	HR	25.1	*0*.*9*	^b^	0.78	*0*.*03*	^b^	3.1	*0*.*2*		0.96	*0*.*07*	^b^	3.8	*0*.*6*	35.3	*20*.*1*	
mid	control	31.9	*2*.*8*		1.07	*0*.*10*		3.4	*0*.*5*		1.43	*0*.*21*		4.6	*0*.*5*	33.0	*64*.*7*	^a b^
	LR	28.9	*1*.*0*	**	1.03	*0*.*03*	**	3.6	*0*.*3*		1.08	*0*.*07*		3.7	*0*.*7*	21.0	*22*.*0*	^a^
	HR	26.6	*0*.*9*		0.96	*0*.*03*	**	3.6	*0*.*2*	*	1.15	*0*.*07*		4.3	*0*.*4*	126.6	*21*.*2*	^b^**
late	control	27.7	*1*.*4*		1.04	*0*.*05*		3.7	*0*.*2*		1.21	*0*.*11*		4.3	*0*.*5*	81.7	*32*.*3*	
	LR	27.3	*1*.*0*	*	1.00	*0*.*04*	**	3.6	*0*.*3*		1.04	*0*.*08*		3.8	*0*.*6*	22.9	*23*.*9*	
	HR	28.7	*0*.*9*	**	1.06	*0*.*03*	**	3.7	*0*.*1*	*	1.22	*0*.*07*	**	4.3	*0*.*4*	62.6	*21*.*2*	

The lactation stages are defined as follows: “early” for ≤ 100 days in lactation (DiL), “mid” for 101 up to 200 DiL, “late” for ≥ 201 DiL. The groups compared are the control group (n = 30), LR (low responder, n = 4) and HR (high responder, n = 5). Milk yield is specified as energy corrected (ECM). The mapped values, including the SCC, are presented as LSM ± SD in the respective lactation stage. The superscripted letters (^a, b^) characterize significant differences (P < 0.05) between groups within the same lactation stage. Asterisks indicate significant differences (*; P < 0.05) or highly significant differences (**; P < 0.01) over time with regard to the early lactation stage within the same group.

### *C*. *diff*. specific and total IgA

**Fig 1:**
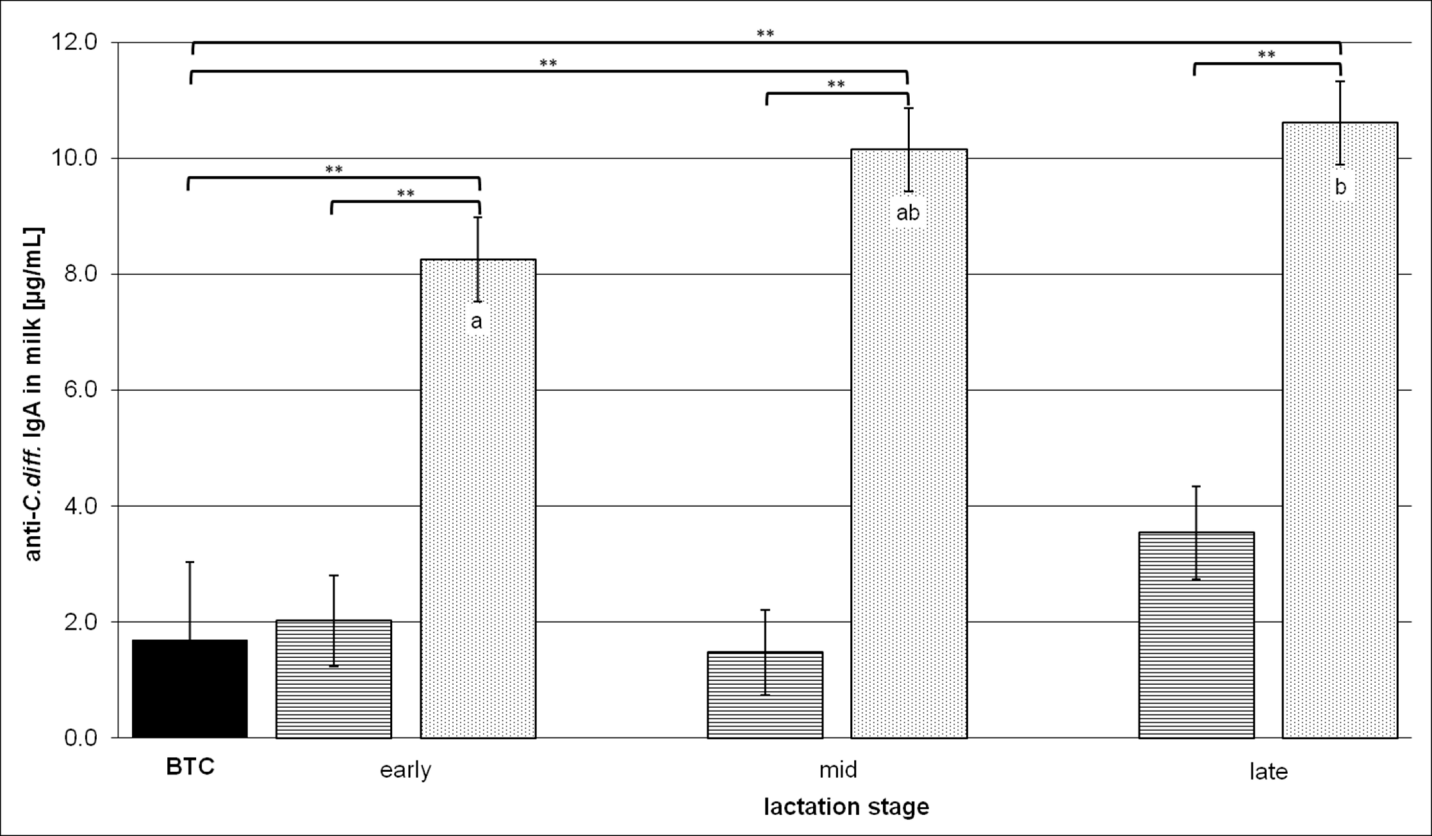
Anti-*C*. *diff*. milk IgA contents in the different lactation stages. BTC means the "before treatment control" value of all cows in frame for vaccination. The lactation stages are defined as “early” for ≤ 100 DiL, “mid” for 101–200 DiL and “late” for ≥ 201 DiL. On the ordinate, *C*. *diff*. specific milk IgA concentrations are depicted as LSM ± SD and presented comparatively for the BTC (n = 9, black bar), low responder (LR, n = 4, cross-striped bars) and high responder (HR, n = 5, dotted bars) groups. Substantial or highly significant differences versus BTC and between LR and HR in the same lactation stage are marked by brackets with one (P < 0.05) or two asterisks (P < 0.01). Significant differences within the same group related to the early lactation stage are shown in lower case letters (a, b) for P < 0.05.

The cows in frame for vaccination showed a baseline value (BTC) of 1.7 ± 1.3 μg/mL anti-*C*. *diff*. IgA in milk at TW 0 (Fig 1), which indicated a natural confrontation with the omnipresent germ *C*. *diff*.. At this initial point in time, milk samples included *C*. *diff*. specific IgA up to 5.1 μg/mL, and the HR cows tended to have more milk anti-*C*. *diff*. IgA than the LR cows (Table 4). *C*. *diff*. specific as well as total IgA in milk were analyzed to compare them with the three lactation stages of the LR and HR groups (early, mid- and late lactation) in order to assess a possible concentration of the milk IgA during the course of lactation. At this point, the anti-*C*. *diff*. IgA milk contents were examined with regard to the BTC, among LR and HR in the same lactation stage, and within the same group compared with the early lactation stage (Fig 1). Compared to BTC, the HR cows had approximately 80% more anti-*C*. *diff*. IgA in milk during any lactation stage, whereas no enrichment of this Ig was found in the milk of the LR group. Accordingly, the amounts of anti-*C*. *diff*. milk IgA produced by the HR group in each lactation stage were significantly higher than those of the LR group. With a view to the continuing lactation and treatment, an increase of 2.4 ± 1.0 μg/mL anti-*C*. *diff*. IgA in HR milk was calculated by the SAS/STAT software starting from 8.3 ± 0.7 μg/mL in early lactation and achieving 10.6 ± 0.7 μg/mL in late lactation.

**Table 4:** Anti-*C*. *diff*. and total IgA contents in milk and blood of treated cows when the initial period (TW 0) and prior to the *MucoCD-I* vaccine batch B was used for the first time (TW 16).

Group	TW	anti-*C*. *diff*. IgA (μg/mL)						total IgA (μg/mL)					
		in milk			in blood			in milk			in blood		
		LSM	*SD*		LSM	*SD*		LSM	*SD*		LSM	*SD*	
**LR**	**0**	0.0	*1*.*6*	* ^(TW 4)^	3.6	*2*.*4*	** ^(TW 6,7)^	73.1	*46*.*2*	** ^(TW 10)^	119.2	*70*.*8*	** ^(TW 3)^
**HR**	**0**	3.8	*2*.*3*	* ^(TW 3, 4)^ ** ^(TW 5)^	6.7	*3*.*4*		133.6	*65*.*3*	** ^(TW 4)^ * ^(TW 14)^	257.8	*100*.*2*	** ^(TW 3, 4)^
**LR**	**16**	1.2	*1*.*6*	^a^	3.6	*2*.*4*		128.6	*46*.*2*		138.0	*70*.*8*	
**HR**	**16**	8.1	*1*.*5*	^b^ ** ^(TW 18)^ * ^(TW 20)^	7.8	*2*.*1*	* ^(TW 18)^	274.7	*41*.*3*	** ^(TW 20, 27)^	223.2	*63*.*4*	

All IgA data are displayed as LSM ± SD group-related to LR (low responder, n = 4) and HR (high responder, n = 5) in different treatment weeks (TW). The superscripted letters (^a, b^) indicate significant differences (P < 0.05) between HR and LR group within the same TW. Asterisks indicate significant differences between defined points in time towards TW 0 or TW 16 with P < 0.05 (*) or P < 0.01 (**).

**Fig 2:**
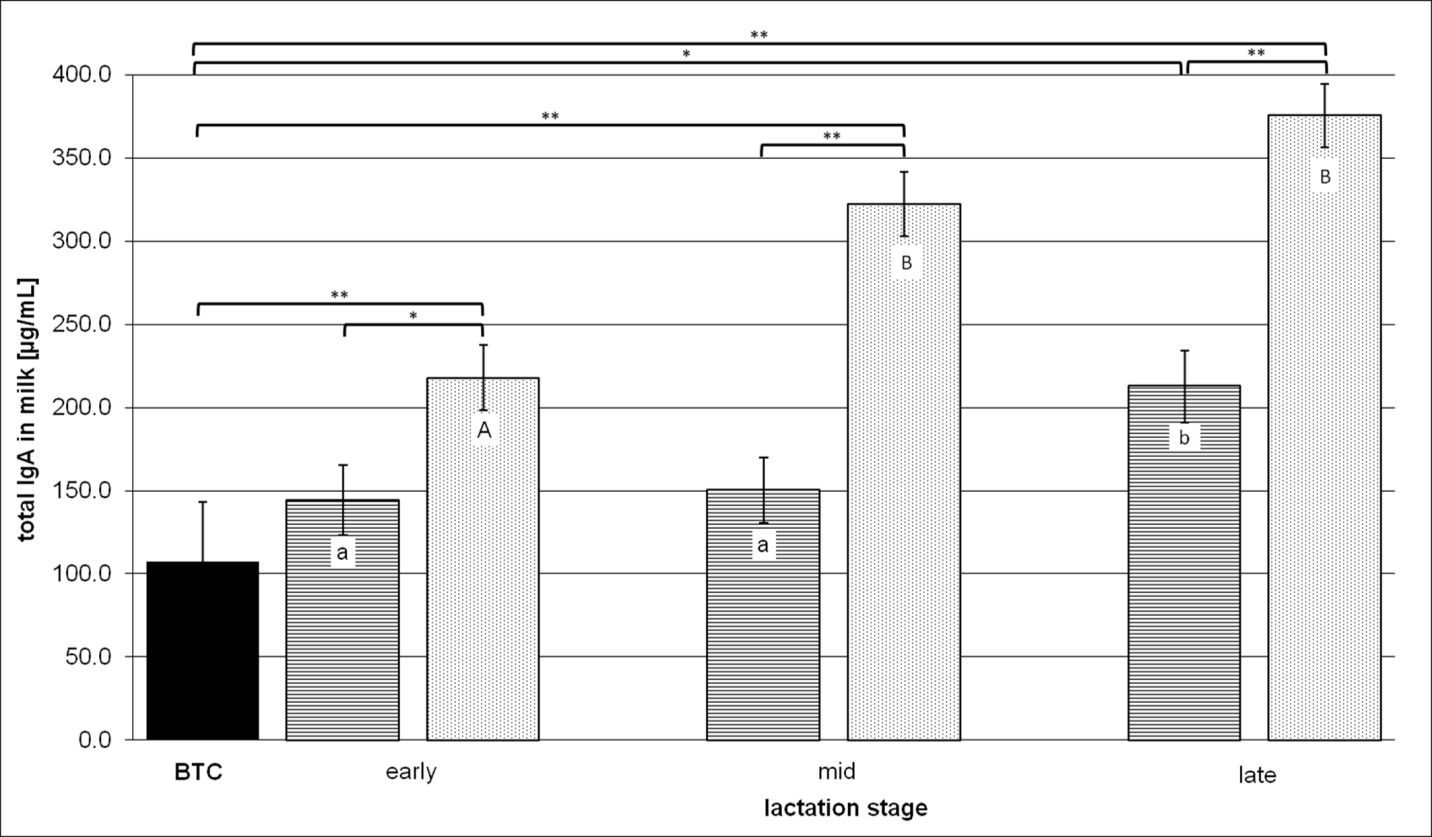
Total milk IgA contents in the different lactation stages. BTC means the "before treatment control" value of all cows in frame for vaccination. The lactation stages are defined as “early” for ≤ 100 DiL, “mid” for 101–200 DiL, “late” for ≥ 201 DiL. The ordinate values represent comparatively LSM ± SD of the BTC (n = 9, black bar), low responder (LR, n = 4, cross-striped bars) and high responder (HR, n = 5, dotted bars) groups. Brackets with asterisks link treated groups with BTC and during the same lactation stage, if significant differences between them are indicated by P < 0.05 (*) or P < 0.01 (**). Significant differences within the same group related to the early lactation stage are indicated by lower case letters (a, b) in case of P < 0.05 and upper case letters (A, B) for P < 0.01.

In accordance with the anti-*C*. *diff*. IgA contents of milk, the same aspects were reconsidered when the total milk IgA contents were evaluated during the three lactation stages (Fig 2). The BTC of total milk IgA was 107.5 ± 35.9 μg/mL, but the total IgA values related to the treated groups differed by nearly the factor of two in TW 0 as indicated in Table 4. This imbalance of the total IgA amounts among milk of HR and LR at the outset of treatment increased by the treatment in the further course of lactation. The HR cows produced an average of 217.8 ± 19.5 μg of total IgA per mL milk in early lactation, two times more than the BTC and roughly one third more than the LR cows secreted during this early lactation period. The total IgA in HR milk increased further by 48% and 72%, respectively, in the subsequent mid- and late lactation stages. An increase of roughly 47% was determined in LR milk only between the early and late lactation periods, peaking at 212.7 ± 21.6 μg/mL. Consequently, this total IgA level in LR cows’ milk also deviated significantly compared to the BTC. The calculation of the relative shares of *C*. *diff*. specific IgA in total milk IgA resulted in 1.6% for the BTC and 1.6% for the LR group in late lactation. The proportion of *C*. *diff*. specific IgA in total milk IgA more than doubled in the HR cows’ early lactation milk by 3.8% towards the BTC, dropping off to roughly 3% during the later lactation stages.

**Fig 3:**
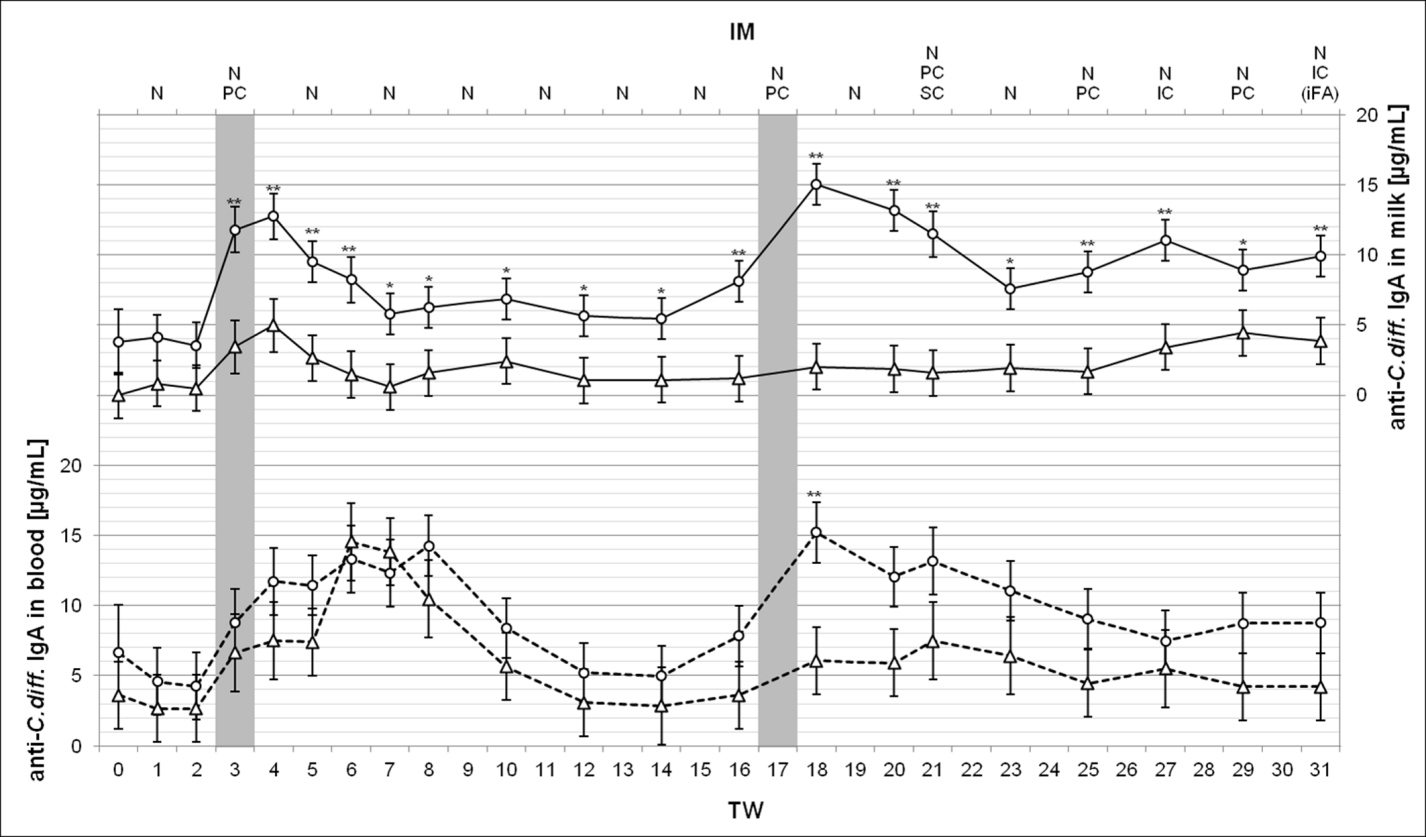
Anti-*C*. *diff*. IgA concentrations in milk and in blood during the 31-week treatment period. The ordinate values in milk (solid lines, top) and in blood (broken lines, bottom) are shown as LSM ± SD per treated groups, the low responder (LR, n = 4, triangles) and the high responder (HR, n = 5, circles). Substantial or highly significant differences between both groups at the same point in time are marked by one (P < 0.05) or two asterisks (P < 0.01). The gray bars indicate the one-time application of *MucoCD-I* vaccine batch A in TW 3 and the first use of vaccine batch B for injection in TW 17. The vaccination routes were nasal (N), percutaneous (PC), subcutaneous (SC) and intracutaneous (IC). The injected *MucoCD-I* vaccine was uniquely supplemented with incomplete Freund’s adjuvant (iFA).

**Fig 4:**
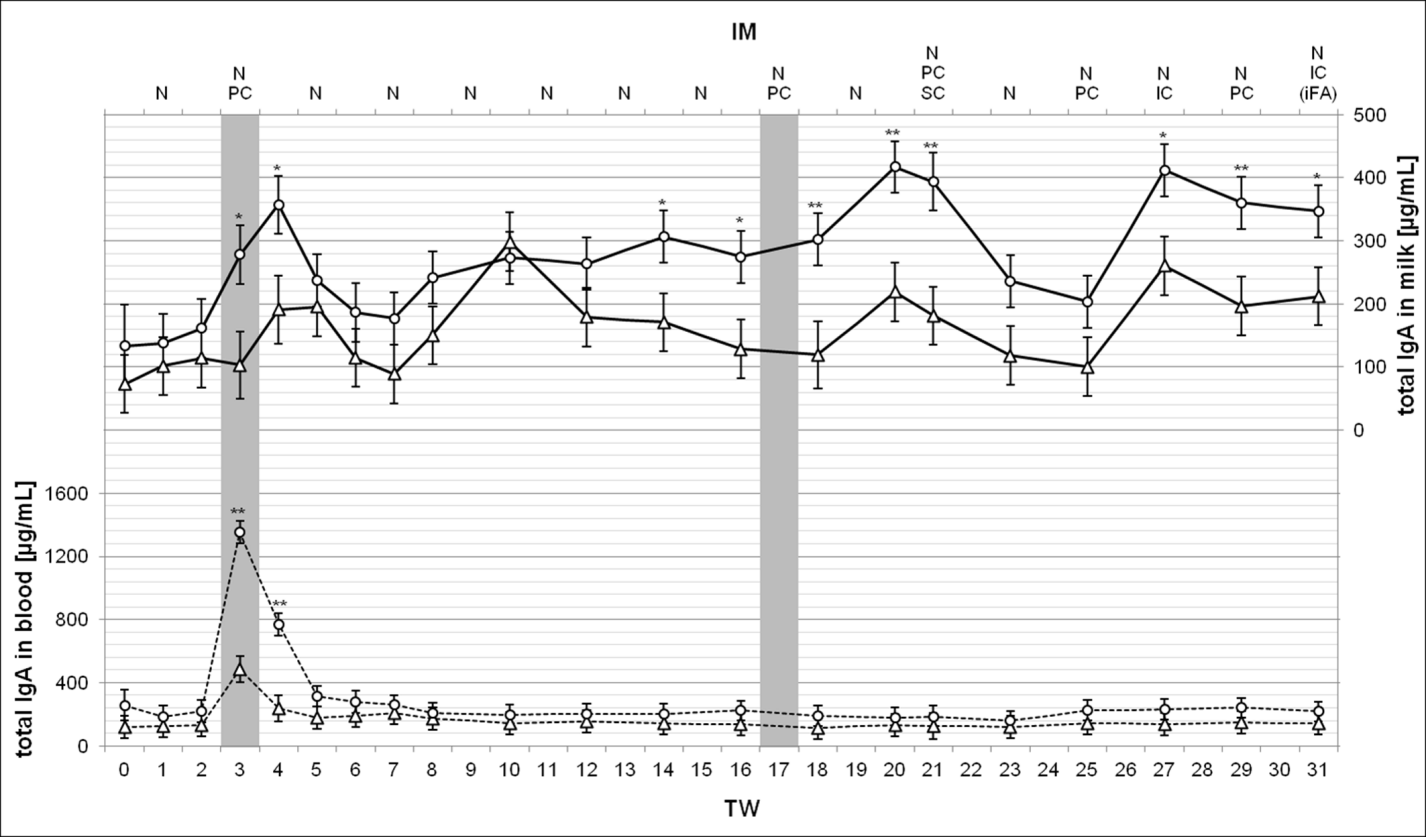
Total IgA concentrations in milk and in blood during 31-weeks treatment period. Measured total IgA values in milk (solid lines, top) and in blood (broken lines, bottom) are shown as LSM ± SD per treated groups, the low responder (LR, n = 4, triangles) and the high responder (HR, n = 5, circles), on the ordinate. Substantial or highly significant differences between both groups compared to the same point in time are marked by one (P < 0.05) or two asterisks (P < 0.01). The gray bars indicate the one-time application of *MucoCD-I* vaccine batch A in TW 3 and the first use of vaccine batch B for injection in TW 17. The vaccination routes were nasal (N), PC, SC and IC. The injected *MucoCD-I* vaccine was uniquely supplemented with iFA.

During the treatment, two different *MucoCD-I* vaccine batches, named batch A and B, were tested. Additionally, the well-tolerated vaccine batch B was administered on various routes. To evaluate in detail the *C*. *diff*. specific and total IgA concentrations in milk and blood possibly changed by the treatment, both parameters were analyzed close-meshed in these body fluids of the treated animals (Figs 3 and 4). Table 4 includes the initial amounts of the measured Igs before the treatment started. Obviously, regarding the investigated antibodies, no significant differences were determined in each body fluid between LR and HR cows at this point in time (TW 0). In terms of *C*. *diff*. specific IgA production in milk, a radical increase resulted from the vaccine batch A given once PC for both treated groups. The value for the HR group initially measured more than tripled in TWs 3 and 4. With approximately 5.0 μg/mL *C*. *diff*. specific IgA in milk, the LR group achieved a substantially different level against TW 0 in TW 4. Following the vaccination in TW 3, all subsequent analysis of the *C*. *diff*. specific IgA in milk showed significant differences between HR and LR. They flattened during the pure N application of vaccine, but increased again after IM with vaccine batch B in TW 16 due to the strong responsiveness of the HR cows. Their *C*. *diff*. specific IgA production peaked at 15.1 ± 1.5 μg/mL in milk in TW 18. This top value and the following in TW 20 were substantially higher than the measured values that were found during the previously N IM period. The *C*. *diff*. specific IgA level in HR milk caused by the first PC injection of vaccine batch B could not be maintained. No later than in TW 23, the level reached was at least 30% lower, and then maintained until the end of treatment, unaffected by other administration routes for the vaccine. The *C*. *diff*. specific IgA contents of the HR group monitored in the blood showed a similar development. Compared with the measured values in HR milk, the specific antibody content of blood increased more slowly after the PC administered vaccine batch A. However, the time period with more than 10 μg/mL *C*. *diff*. specific IgA in blood was prolonged and ended with the highest value of 14.3 ± 2.1 μg/mL in TW 8. The vaccine batch B given helped to boost this antibody once again to 15.2 ± 2.1 μg/mL in TW 18. Only then did the *C*. *diff*. specific IgA level in HR blood differ from that in LR blood. During the remaining treatment period, a slight decrease to the primary level in HR blood was noticed. The *C*. *diff*. specific IgA level in LR blood tended to be lower than that of its counterpart, apart from TWs 6 and 7. Only in these TWs, the measured values of approximately 15 μg/mL *C*. *diff*. specific IgA in LR blood were significantly higher than the initial values, reaching 3.6 ± 2.4 μg/mL (Table 4). The use of vaccine batch B showed no similar effects.

The analysis of total IgA concentrations in milk (Fig 4) revealed results similar to *C*. *diff*. specific IgA concentrations regarding their development over the complete treatment period. The HR group data showed once again a rapid increase after injecting vaccine batch A in TW 3, a slowdown during the pure N IM, and a renewed increase after vaccine batch B was administered. The first IC injection of vaccine batch B in TW 27 induced increased total IgA in milk, which was on the same level, as measured in TWs 20 and 21. With about 400 μg/mL, the HR cows produced three times more than when treatment started. Regarding the dates of the top values determined in HR milk, the differences with regard to the LR group were significant when comparing the total IgA milk contents. The range of total IgA in LR milk was between 70 and 360 μg/mL. Overall, total IgA secretion by LR seemed to be unaffected by the treatment. The cause of the outlier value recorded in TW 10 remained unknown. Except for the effect of vaccine batch A on HR cows, the total IgA concentrations of HR and LR did not differ in the blood. Consequently, the blood serum contents of both groups for total IgA clearly peaked only in TWs 3 and 4 even though the LR blood contained four times more total IgA, whereas the HR cows reached a fivefold increase.

### Correlation analysis

The calculated Pearson correlation coefficients were classified as follows: *I r I < 0*.*5* as “weak”, *0*.*5* *≤* *I r I < 0*.*8* as “middle” and *I r I* *≥* *0*.8 as “strong” relations between the examined variable pairs. Apart from the milk yield, all other variables summarized in Table 5 showed positive linear dependency in milk referring to *C*. *diff*. specific and total IgA. Primarily, weak relations were determined between the pairs of variables that were the subject of the research. Nevertheless, total IgA concentrations tended to be stronger depending on some factors like duration of lactation (marked by DiL) and major milk components, milk proteins and fats, than the *C*. *diff*. specific IgA content of milk. This difference in the specific and total Igs dependency on the above-mentioned dairy production suggests that these factors could be caused by the vaccination schedule, which aimed at the enrichment of *C*. *diff*. specific IgA in milk. Additionally, total IgA content was more affected by the natural concentration of milk components in the late lactation stage. Due to the correlation analysis, the relationship between *C*. *diff*. specific IgA and total IgA contents could only be classified as moderate. Compared with the weaker dependencies on the other variables, this result was not surprising because *C*. *diff*. specific IgA presented some of the total IgA as well.

**Table 5:** Correlations between anti-*C*. *diff*., total IgA and the main production factors in milk of treated cows (n = 9).

(n = 165)	anti-*C*. *diff*. IgA (μg/mL)		total IgA (μg/mL)	
	*r*	P	*r*	P
DiL	*0*.*19*	< 0.05	*0*.*37*	< 0.01
ECM yield (kg/d)	*-0*.*25*	< 0.01	*-0*.*30*	< 0.01
milk protein (kg/d)	*0*.*27*	< 0.01	*0*.*47*	< 0.01
milk fat (kg/d)	*0*.*30*	< 0.01	*0*.*44*	< 0.01
SCC (x1,000/mL)	*0*.*19*	< 0.05	*0*.*21*	< 0.01
total IgA (μg/mL)	*0*.*69*	< 0.01	-	-

“*r*” indicates the Pearson product moment correlation coefficient. Significant correlations between the investigated variables must be proven by P < 0.05 and highly significant correlations are assessed with P < 0.01. Positive *r* displays pairs of variables tending to increase linearly together. For the pairs with negative *r*, one variable tends to develop in an inversely proportional way. Abbreviations used for some variables are: *DiL* for days in lactation, *ECM yield* for energy corrected milk yield, and *SCC* for somatic cell counts. The evaluated number of samples (n) was 165.

## Discussion

The present study aimed to achieve the enrichment of *C*. *diff*. specific antibodies, preferably sIgA in mature cow's milk, based on the similar vaccination schedule that has proven to be effective earlier [15]. The different vaccines used for IM against *C*. *diff*. were well tolerated by the treated cows apart from the *MucoCD-I* vaccine batch A, when their monitored health was taken into account. *MucoCD-I* vaccine batch A contained excessive amounts of TcdA and TcdB as a vaccine production-related fault. Its one-time administration implicated pro-inflammatory effects in the treated cows due to the remaining biological activity of these potent exotoxins. Consequently, several vaccinated animals fell ill in TW 3, and the administration of antigen via injection had to be discontinued until a newly composed *MucoCD-I* vaccine was available. To maintain the stimulation of the treated cows' IS with *C*. *diff*., the bi-weekly N IM was steadily carried out assuming the concept of the “common mucosal IS” [25]. Thus, the IM of the NALT against *C*. *diff*. should also evoke a humoral immune response in the mammary gland. Nevertheless, the systemic IM on parenteral routes was pursued with *MucoCD-I* vaccine batch B to trigger a cumulative antibody response in peripheral lymphoid tissues. Following treatment with *MucoCD-I* batch B when PC was given for the first time, a new dynamic in production of *C*. *diff*. specific IgA was achieved as measured, especially, in the milk and blood of the HR group (Fig 3). However, the specific antibody levels within the range of 15 μg per mL milk were unachievable, neither on the SC nor on the IC route. The humoral immune response of the treated animals was also evaluated depending on the indirect measurement of serum IgA. This approach is known to be problematic because it does "not necessarily reflect the effector response at the mucosal surface" [26]. Even though the treatment influenced the development of the anti-*C*. *diff*. IgA concentrations in blood serum, the mucosal immune response should not assessed solely with the specific antibodies, considering the LR immune response during the pure N treatment period.

Regarding treatment effects on the milk yield and its composition, the *MucoCD-I* vaccine batch A applied in TW 3 caused a reduced daily milk output, including the total quantities of milk fats and proteins within early lactation (Table 3). Due to the harmful effect of this vaccine, the treated cows' general condition was impaired and their milk performance reduced. Health and performance are closely interrelated throughout lactation [27]. Apart from *MucoCD-I* vaccine batch A given in TW 3, all other anti-*C*. *diff*. vaccinations in this study had no impact on any cow health parameter, milk production and macronutrient composition. These findings were in accordance with study results of Gingerich et al. (2008), exploring the safety of milk ingredients from hyperimmunized cows with a multivalent bacterin (S100) [28]. In the study referred to, the specificity but not the total amount of the evaluated milk antibodies was affected by the S100 treatment [28]. Another *C*. *diff*. IM study showed significantly increased antibody concentrations against *C*. *diff*. and its exotoxins in raw milk [29]. In contrast to the present study, Young et al. (2007) did not release the development of the antigen-specific sIgA production during the IM period. Their mean value disclosed for anti-*C*. *diff*. sIgA in raw milk was 16 μg/mL on average [29]. Hereinafter, it was used as reference for discussing the outcomes, as shown in Figs 1 and 3, because the IM procedure applied against *C*. *diff*. was similar, and the ELISA was also used as measuring method to ascertain the specific Ig milk contents. The reference value for *C*. *diff*. specific sIgA revealed by Young et al. (2007) was comparatively higher than 10 to 11 μg/mL of the specific milk antibody produced by the HR group during mid- and late lactation (Fig 1). The interrupted vaccination on parenteral routes following TW 3 may have led to this minor amount. However, the following facts should be respected when making that assessment. Firstly, the individual immune responsiveness of the treated animals varied dramatically as seen, for example, in the two extremes in the last two thirds of lactation: HR-cow 8 produced on average 15 μg/mL of *C*. *diff*. specific sIgA, whereas LR-cow 2 did not even reach 1 μg/mL. Secondly, the dairy cow breed *Brown Swiss* used for immune milk production could basically be less sensitive to immune challenges than other breeds due to stronger innate immune defense mechanisms. For instance, the *Holstein Frisian* with a history of selection for milk yield at the expense of disease resistance [30] may trigger a more powerful humoral immune response. In principle, colostral Ig concentrations were proven to be as variable between dairy breeds [31]. Likewise, such variations may be present in mature milk due to the genetic differences of the investigated breeds. Thirdly, the age or the number of elapsed lactations shape the immunological status of mammalians, as measured exemplarily by amounts of bovine colostral Igs [32]. Finally, the vaccine used for triggering the immune response probably had a different composition, e.g. included adjuvants. Neither van Dissel et al. (2005) nor Young et al. (2007) disclosed the breed of the immunized dairy cattle and the vaccine composition that was used [15, 29]. In contrast to these previous IM studies, the study at hand highlights the achievable quantities of anti-*C*. *diff*. IgA in bovine milk during the progressing lactation following sustained immune stimulation of dairy cows. The potential output is important knowledge for every dairy producer intending to commercialize the anti-*C*. *diff*. IgA production in bovine milk as a dietary supplement for human health. The necessarily qualitative assessment of the anti-*C*. *diff*. IgA produced in milk is still pending.

No direct comparison could be made between the total milk IgA measurements presented and the same ones obtained by Young et al. (2007) due to different analytical approaches [29]. As depicted in Fig 2, the vaccinated animals classified by their different immune responsiveness showed a sharp contrast between the group-related total IgA contents. That was not expected as resulting from the *C*. *diff*. specific IM, considering that the secreted *C*. *diff*. specific IgA was only slightly less than 5% of total IgA in milk. Cross-reactions in antibody production of various sizes may have contributed to different increases of total IgA when LR and HR are compared. In addition, fundamental differences between the number of APCs in mammary tissues or the mammary epithelial placements with the IgA transport mediating specific receptors (pIgR) might be responsible for the varying immune responsiveness of the treated cows. The concentrations of all milk Igs were shown to increase during late lactation, which coincides with a major reduction in milk yield [33]. This result parallels the weak inverse relation between anti-*C*. *diff*. or total IgA and milk yield as calculated in the present study (Table 5), whereas the correlation analysis of all other production factors investigated and anti-*C*. *diff*. or total milk IgA revealed a slightly positive interdependency. For example, the SCC was found to be a factor with strong significant correlation to total milk IgA concentration [34]. An elevated SCC is often accompanied by increased proportions of a few whey proteins, including Igs [35]. Furthermore, milk IgA concentrations are obviously associated with the stage of lactation [34] that was reproduced by the examined correlation to DiL. The dependency could only be described as weak, presumably due to the sample size that was considerably smaller in the available study as opposed to the comparative study [34]. Given the fact that total IgA also covers anti-*C*. *diff*. IgA and that this whey protein is, in turn, part of total milk proteins, the closely commutated linkages of these variables could be verified by the results of the correlation analysis (Table 5). Phenotypic correlations among milk constituents are known to be strongly positive [36], regardless of the significant differences between their proportions among breeds and parities [37]. Accordingly, anti-*C*. *diff*. and total milk IgA as elements of total milk protein content are also related to the daily milk fat amount. Notwithstanding the close knitted relation of anti-*C*. *diff*. to total milk IgA (*r = 0*.*69*), the correlations between the *C*. *diff*. specific IgA and the production factors were generally weaker than the findings for total IgA.

## Conclusion

To conclude, a lasting *C*. *diff*. specific IgA enrichment in milk can be achieved by continuous IM of dairy cows based on two conditions. Firstly, a potent vaccine including the pivotal virulent factors of *C*. *diff*. is crucial. However, a poorly effective vaccine cannot be compensated by the IM procedure, which is carried out to induce a more mucosa-related immune response. Secondly, dairy cows sensitive towards *C*. *diff*. should be preselected as measured by their *C*. *diff*. specific milk IgA. Factors possibly causing different immune responsiveness might be intramammary variations given the prevalence of immune cells like APCs, differences between intercellular signaling following the IM or differences between the epithelial IgA transport capacities depending on the pIgR. These topics will be addressed in a further publication.
